# Lithium Enhances Axonal Regeneration in Peripheral Nerve by Inhibiting Glycogen Synthase Kinase 3****β**** Activation

**DOI:** 10.1155/2014/658753

**Published:** 2014-05-20

**Authors:** Huanxing Su, Qiuju Yuan, Dajiang Qin, Xiaoying Yang, Wai-Man Wong, Kwok-Fai So, Wutian Wu

**Affiliations:** ^1^State Key Laboratory of Quality Research in Chinese Medicine, Institute of Chinese Medical Sciences, University of Macau, Macau; ^2^Department of Anatomy, Li Ka Shing Faculty of Medicine, The University of Hong Kong, Pokfulam, Hong Kong; ^3^GHM Institute of CNS Regeneration, Jinan University, Guangzhou 510632, China

## Abstract

Brachial plexus injury often involves traumatic root avulsion resulting in permanent paralysis of the innervated muscles. The lack of sufficient regeneration from spinal motoneurons to the peripheral nerve (PN) is considered to be one of the major causes of the unsatisfactory outcome of various surgical interventions for repair of the devastating injury. The present study was undertaken to investigate potential inhibitory signals which influence axonal regeneration after root avulsion injury. The results of the study showed that root avulsion triggered GSK-3**β** activation in the injured motoneurons and remaining axons in the ventral funiculus. Systemic application of a clinical dose of lithium suppressed activated GSK-3**β** in the lesioned spinal cord to the normal level and induced extensive axonal regeneration into replanted ventral roots. Our study suggests that GSK-3**β** activity is involved in negative regulation for axonal elongation and regeneration and lithium, the specific GSK-3**β** inhibitor, enhances motoneuron regeneration from CNS to PNS.

## 1. Introduction

Over 50 years ago, lithium was discovered to be efficacious in treatment of bipolar disorders and it remains one of the primary antidepressant drugs. Recently, accumulating evidence suggests that lithium has more significant clinical implications for treating neurological disorders including Alzheimer's disease, Parkinson's disease, ischemic brain injury, Huntington's disease, and amyotrophic lateral sclerosis because of its potent neuroprotective and neurogenesis-promoting ability [1–5]. Moreover, it has been shown that treatment with a clinical dose of lithium to rats with thoracic spinal cord transaction of contusions injuries induces significant descending corticospinal and serotonergic axon regeneration and promotes locomotor functional recovery [6], indicating that lithium can be applied to treat traumatic injury to the spinal cord. With the development of research work into lithium's actions, it is believed that lithium application can be expanded to more neurological diseases.

Although it is believed that motoneurons can regenerate following peripheral nerve injury, the number of regenerating motoneurons is minimal and is not enough for full functional recovery. Brachial plexus injury often involves avulsion of several nerve roots from the cervical spinal cord, leading to massive motoneuron death and permanent paralysis of the innervated muscles [7–10]. Various approaches focusing on microsurgical interventions have been extensively studied to restore target innervation and functional recovery after avulsion [11–15]. However, the treatment for the devastating injury is still a challenging clinical and surgical problem. The challenge which lies in the brachial plexus injury treatment is that motoneuron death in the lesioned segments is relatively high and little is known about how inhibitory signals or lack of appropriate guidance molecules influences regeneration.

In the present study, we investigated the potential roles of lithium in treatment of brachial plexus injury with root avulsion. Our study shows that lithium treatment markedly reduced the activation of GSK-3*β* triggered by root avulsion and enhanced dendritic emanation and axonal regeneration of injured motoneurons after ventral root replantation. The results of the present study demonstrate for the first time that root avulsion stimulates the activation of GSK-3*β* in the injured spinal cord and inactivation of GSK 3*β* by lithium treatment has beneficial effects on motoneuron regeneration after brachial plexus injury.

## 2. Materials and Methods

All surgical interventions and subsequent care and treatment were approved by the Committee on the Use of Live Animals for Teaching and Research of the University of Hong Kong.

### 2.1. GSK-3*β* Activity Assays in the Avulsion-Injured Spinal Cord

Twelve adult female Sprague-Dawley rats (220–250 g) were anesthetized with an intraperitoneal injection of ketamine (80 mg/kg) and xylazine (8 mg/kg). Root avulsion was performed as described previously [16, 17]. Briefly, a dorsal hemilaminectomy on the right side of the sixth cervical vertebra was carried out under aseptic conditions. The 7th cervical spinal roots (C7) were avulsed by traction with a fine hook under a surgical microscope. Total avulsion was checked by visual inspection. Immediately after root avulsion, the animals were randomly divided into 2 groups (6 animals in each group) which received either an intraperitoneal injection of lithium chloride (85 mg/kg bodyweight) [18] or saline as the control. Twenty-four hours after injury, all the animals were perfused intracardially with cold 0.01 M PBS for 5 min. To evaluate activated GSK-3*β* signals in the avulsed spinal cord, we directly stained 30 *μ*m cross-sections of fixed C7 segments harvested from the remaining animals with the antibody against p-GSK-3*β* ^Tyr216^. The activity of GSK-3*β* is positively regulated by phosphorylation of Tyr^216^ [19]. Thus the level of p-GSK-3*β* ^Tyr216^ represents active GSK-3*β*.

### 2.2. Root Avulsion, Ventral Root Replantation, and Axonal Tracing

Ventral root reimplantation model was used to investigate potential roles of lithium in treatment of brachial plexus injury with root avulsion. Techniques for restoration of connectivity by reimplantation of avulsed ventral roots have been extensively developed in animal and human models [11–15]. In most of these studies, the ventral root was directly inserted into the spinal cord after an incision was made in the white matter. Although the insertion site had been optimized so as to minimize functional disorder of the spinal cord [14], the possibility of spinal cord injury remained. In order to avoid damaging the spinal cord by incision, we have developed a new microsurgical technique in which the avulsed ventral root was placed on the pial surface of the spinal cord and the avulsed dorsal root was sutured to the edge of the dura mater to fix the repositioned ventral root in place [16, 20]. A total of 27 adult female Sprague-Dawley rats (220–250 g) underwent this ventral root reimplantation microsurgery immediately after the right C7 spinal nerve avulsion. After surgery, the animals were evenly divided into 3 groups (*n* = 9 for each group) in which one group received daily intraperitoneal injection of lithium chloride (85 mg/kg bodyweight), another group received subcutaneous injections of a selective GSK-3 inhibitor, SB415286 (SB) (1 mg/kg/d), with syringes (two times per day) [6], and the third group received saline as the control treatment. Animals were allowed to survive for 6 weeks. Three days before the end of the survival period, we injected 0.5 *μ*L of 3% FluoroGold (FG) into the C7 spinal nerve via a Hamilton syringe with the needle tip sharpened to label the regenerating neurons.

### 2.3. Histology

At the end of the survival period, the animals were killed with a lethal dose of sodium pentobarbital and perfused intracardially with 0.01 M PBS, followed by perfusion with 200–300 mL of fixative solution containing 4% paraformaldehyde in 0.1 M PB. Spinal cords were harvested and postfixed in fresh fixative solution overnight and subsequently placed in 30% sucrose—0.1 M PB at 4°C for 2-3 days. The C7 segment of the spinal cord was cut into 30 *μ*m cross-sections on a microtome (American Optical Company, NY, USA), mounted on the slides, protected by cover slips, and examined under a fluorescence microscope to count FG-positive cells. Only labeled neurons with visible nuclei were counted. Then we quantified the surviving motoneurons according to a previously described method [21, 22]. Briefly, one of every other cross-section, totally 25 sections per animal, was stained with 1% neutral red. Motoneurons were counted on both sides of the spinal cord. Only those nucleolated profiles apparently belonging to motoneurons were counted to avoid duplication. The number of motoneurons on the intact side was expressed as 100% of the control value. The number of surviving motoneurons on the lesioned side was described quantitatively as percentages of the normal control number.

### 2.4. Statistical Analyses

Statistical differences between two groups were determined by two-tailed Student's *t*-test. Multiple group comparisons were made by one-way ANOVA and Tukey post hoc test. Data were presented as mean ± SEM. Significance levels were set to 0.05 for all comparisons.

## 3. Results

### 3.1. Lithium Treatment Suppressed GSK-3*β* Activation Triggered by Root Avulsion

**Figure 1:**
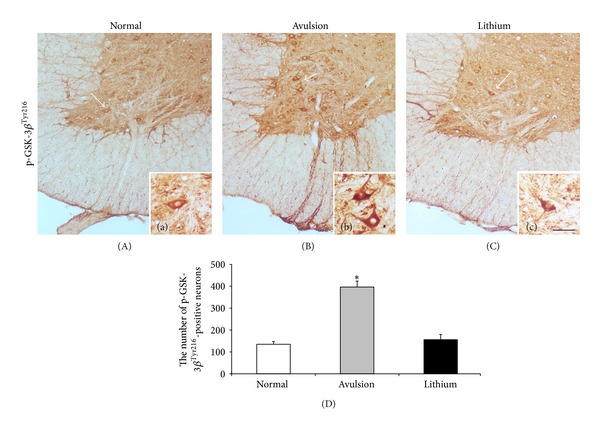
Lithium treatment suppressed GSK-3*β* activation in the avulsion-injured ventral horn. Immunostaining with p-GSK-3*β* ^Tyr216^ on cross-sections of the spinal cord of animals: (A) normal animals, (B) animals which received root avulsion, and (C) animals which received lithium treatment after root avulsion. ((a), (b), (c)) The arrow-pointed areas under higher magnifications. (D) The number of p-GSK-3*β* ^Tyr216^-positive neurons in the ventral horn at 24 h after root avulsion was significantly increased compared with that in the normal ventral horn and treatment with lithium markedly reduced avulsion-induced GSK-3*β* activation to the normal level (*P < 0.001; scale bar: 180 *μ*m in (A), (B), and (C); 40 *μ*m in (a), (b), and (c)).

To assess endogenous alterations of GSK-3*β* activity after avulsion injury, we examined the expression of p-GSK-3*β* ^Tyr216^ using immunostaining. Positive immunoreactivity for p-GSK-3*β* ^Tyr216^ was almost absent in the ventral funiculus and ventral root exit zone in normal animals (Figure 1(A)) and around 135 ± 11.6 p-GSK-3*β* ^Tyr216^-positive neurons were detected in the normal ventral horn (Figure 1(A)). The number of p-GSK-3*β* ^Tyr216^-positive neurons was significantly increased 24 h after root avulsion (397 ± 27.2, Figures 1(B) and 1(D); *P* < 0.001 compared with the normal) and positive immunoreactivity for p-GSK-3*β* ^Tyr216^ was obviously observed along the ventral funiculus as well as in the ventral root exit zone (Figure 1(B)), suggesting that root avulsion triggered the activation of GSK-3*β* in the spinal cord. Treatment with lithium markedly repressed avulsion-induced GSK-3*β* activation to the normal level as shown by a decline in the number of p-GSK-3*β* ^Tyr216^-positive neurons and p-GSK-3*β* ^Tyr216^ immunoreactivity along the ventral funiculus (156 ± 13.5, Figures 1(C) and 1(D); *P* < 0.001 compared with the avulsion group).

### 3.2. Lithium Treatment Increased Dendritic Emanation and Axonal Regeneration of Motoneurons after Replantation of Ventral Roots

**Figure 2:**
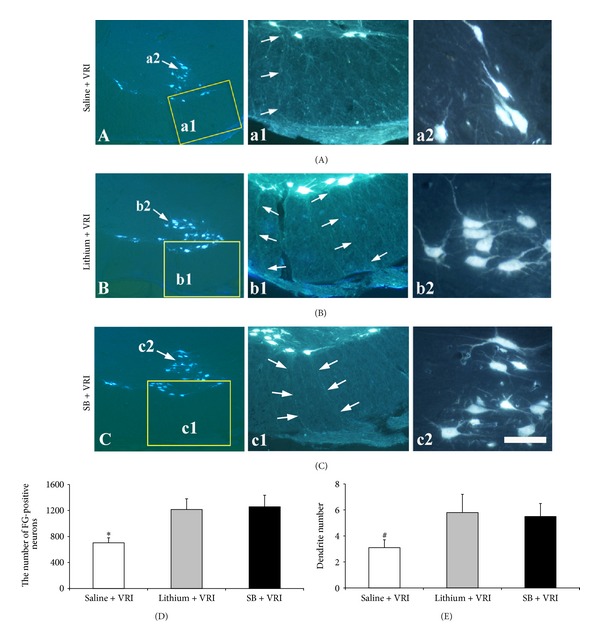
Lithium treatment increased axonal regeneration and dendritic emanation of motoneurons after replantation of avulsed ventral roots. (A) A representative micrograph of spinal cross-sections showing FG-positive neurons (arrows) present in the ventral horn of the animals with ventral root reimplantation (VRI) plus saline treatment as controls. (a1) Micrographs made under higher magnification of the rectangular area in A showing that FG-labeled neurons extended their axons into the replanted ventral roots; (a2) micrographs made under higher magnification of the arrow-pointed area in A showing dendritic emanation of FG-labeled neurons in the ventral horn. (B) A representative micrograph of spinal cross-sections showing FG-positive neurons (arrows) present in the ventral horn of the animals with ventral root reimplantation (VRI) plus lithium treatment. (b1) Micrographs made under higher magnification of the rectangular area in B showing that FG-labeled neurons extended their axons into the replanted ventral roots; (b2) micrographs made under higher magnification of the arrow-pointed area in B showing dendritic emanation of FG-labeled neurons in the ventral horn. (C) A representative micrograph of spinal cross-sections showing FG-positive neurons (arrows) present in the ventral horn of the animals with ventral root reimplantation (VRI) plus SB injection. (c1) Micrographs made under higher magnification of the rectangular area in C showing that FG-labeled neurons extended their axons into the replanted ventral roots; (c2) micrographs made under higher magnification of the arrow-pointed area in C showing dendritic emanation of FG-labeled neurons in the ventral horn. (D) The number of regenerating motoneurons that extended axons into replanted ventral roots in the lithium-treated animals was significantly higher than that in the saline control animals (*P < 0.001). (E) The number of dendritic emanation from regenerating motoneurons in the lithium-treated animals was significantly higher than that in the saline control animals (^#^ *P* < 0.05). Scale bar: 180 *μ*m in A, B, and C; 75 *μ*m in (a1), (a2), (b1), (b2), (c1), and (c2).

During harvesting spinal cords, gross anatomical investigations confirmed that the replanted ventral roots were firmly attached to the ventrolateral aspect of the spinal cord in all the 27 animals. Retrograde labeling with FG showed that regenerating motoneurons extended their axons into the replanted ventral roots attached to the surface of avulsed spinal cord in all of the animals (Figures 2(a1), 2(b1), and 2(c1)). In saline-treated animals, ventral root replantation induced 703 ± 76.5 FG-positive neurons in the C7 ventral horn (Figures 2(A) and 2(C)). Notably, systemic application of lithium led to a dramatic increase in the number of FG-positive neurons present in the C7 ventral horn (1217 ± 163.9, Figures 2(B) and 2(C); *P* < 0.001 compared with the saline group). A dramatic increase in the number of FG-positive neurons in the C7 ventral horn was also observed in the animals which received subcutaneous SB injections (1258 ± 178.2, Figures 2(C) and 2(D); *P* < 0.001 compared with the saline group). Furthermore, both lithium treatment and SB application induced a significant increase in the number of dendritic emanation per FG-positive neuron compared with the saline control (5.8 ± 1.4 versus 3.1 ± 0.6 and 5.5 ± 1.0 versus 3.1 ± 0.6 resp.; *P* < 0.05, Figures 2(a2), 2(b2), 2(c2), and 2(E)).

**Figure 3:**
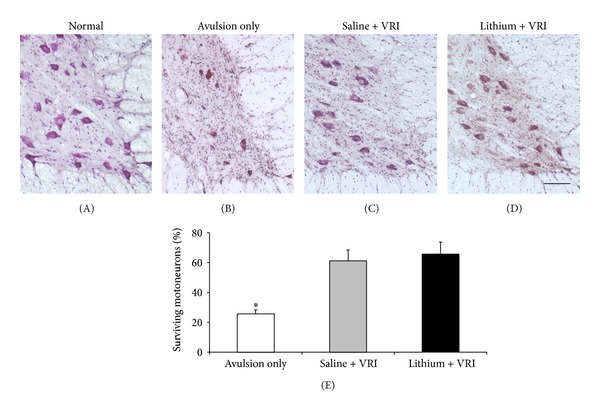
Effects of lithium treatment and ventral root reimplantation (VRI) on the survival of host motoneurons as revealed by neutral red staining 6 weeks after root avulsion. (A) Normal animals. (B) Animals receiving root avulsion only. (C) Animals receiving ventral root reimplantation (VRI) plus saline injection. (D) Animals receiving ventral root reimplantation (VRI) plus lithium treatment. (E) VRI significantly increased the survival rate of motoneurons compared to controls (*P < 0.001 compared to root avulsion only). Lithium treatment did not further increase the motoneuron survival after VRI. There were no statistically significant differences in the survival rate of motoneurons between saline- and lithium-treated animals. Scale bar: 150 *μ*m.

We then investigated the effect of lithium on motoneurons survival after ventral root replantation. Six weeks after root avulsion, only 25.6 ± 2.8% of motoneurons survived in the ventral horn in contrast to the normal side (Figures 3(A) and 3(B)). Root ventral replantation significantly increased the number of surviving motoneurons. In saline-treated animals, 61.2 ± 7.3% of motoneurons survived 6 weeks after ventral root replantation, which is significantly higher than that in animals which received avulsion only (*P* < 0.001, Figures 3(C) and 3(E)). In contrast to promoting effects of lithium on regeneration, lithium treatment did not further increase the motoneuron survival after ventral root replantation (65.4 ± 8.1%, Figure 3(D)). There were no statistically significant differences in the survival rate of motoneurons between saline- and lithium-treated animals (*P* > 0.05, Figure 3(E)).

## 4. Discussion

Microsurgical interventions with ventral root reimplantation have been widely used to restore target innervation and functional recovery after brachial plexus injury. However, the overall outcome of these surgical strategies with respect to the entire arm function remains poor. A possible reason is that the number of regenerating motoneurons which send out axons to innervate peripheral targets is not sufficient to achieve a significant recovery of function. It has been suggested that a combination of various approaches may be more effective for motoneuron survival and axonal regeneration in the treatment of avulsion lesions of the spinal cord [8]. Therefore, various combinatory strategies have been adopted in animal models to test their efficiency in treating root avulsion injury. It was reported that BDNF treatment significantly improved the survival of injured motoneurons and enhanced the regrowth axon sprouts into the distal stump of musculocutaneous nerve in a C5 ventral root avulsion-reimplantation rat model [23]. Single dose application of CNTF and BDNF improved remyelination of regenerating nerve fibers after C7 ventral root avulsion and replantation [24]. Cotreatment with riluzole and GDNF showed significantly improved locomotor function accompanied with the increase in the number of surviving and regenerating motoneurons after ventral root avulsion injury [25]. However, the difficulty in the administration of neurotrophic factors and their short half-life period limit their application in treating CNS traumatic injury. Recently cell replacement therapies have been applied to treat neurological disorders and a variety of stem cells have been transplanted into the ventral horn to treat spinal motoneuron degeneration and denervation [26–29]. Although some promising phenomena have been found in these studies, it is still a tremendous challenge for transplanted cells to integrate precisely within the complex host circuit and find a way to grow axons within the lesioned spinal cord into the replanted root.

A direct and efficient treatment for root avulsion injury is to induce sufficient regeneration of injured motoneurons. The unfavorable microenvironment of injured spinal cords is a major obstacle for significant regeneration. The problem which lies in the treatment for root avulsion injury is that we know little about how inhibitory signals present in the avulsed spinal cord influence regeneration. Previous studies reported that avulsion injury stimulated an activation of astrocytes, microglia, and macrophages [30, 31], which may play a negative role in axonal regeneration. Our study shows that root avulsion triggers an activation of GSK-3*β* within 24 hours. This is the first demonstration that the expression of activated GSK-3*β* is elevated in the avulsed spinal cord, especially in the injured motoneurons and along the ventral funiculus. It has been reported that GSK-3*β* activation induces the collapse of growth cones of cultured neurons and suppresses their axon formation [32, 33]. It has also been shown that axon growth inhibitors such as chondroitin sulfate proteoglycans (CSPG) and semaphorins repel axon extension by activating GSK-3*β* [6, 34, 35]. Therefore, GSK-3*β* activity is supposed to be closely involved in influencing axon elongation of CNS neurons. Several studies have demonstrated that pharmacological inhibition of GSK-3*β* activity results in enhanced axonal growth both* in vitro* and* in vivo* [6, 36, 37], suggesting that regulating GSK-3*β* activity may be a prominent target to improve regeneration after CNS injury. Our present study shows that systemic application of lithium, a specific GSK-3*β* inhibitor, significantly suppresses GSK-3*β* activation triggered by root avulsion in the injured spinal cord and increases axonal regeneration of avulsed motoneurons into replanted ventral roots. In addition, more dendrites are found to emanate from regenerating neurons in lithium-treated animals with replanted ventral roots. The results of our study provide evidence for the first time that lithium has therapeutic potentials in treatment with brachial plexus injury due to its potent effects on promoting axonal regeneration into replanted ventral roots. Interestingly, lithium treatment shows no effects on the survival of avulsed motoneurons, suggesting that GSK-3*β* activity is not a critical regulator for the survival of injured motoneurons.

Lithium is widely used in humans as an antidepressant drug. Its administration is easy and convenient. These indicate the prospects of applying lithium to treat brachial plexus injury. However, it should be noted that even if extensive axonal regeneration has been induced, it is still far away from the success of healing the devastating injury. Regenerating axons need an extensive amount of time to reach targets and they have to be functionally competent to reinnervate the paralyzed muscles. Can lithium treatment promote elongation speed of regenerating axons? Can lithium treatment improve functional competency of regenerating axons and eventually lead to satisfactory recovery of function? A series of experiments addressing these issues are warranted in the coming future in order to fully evaluate the therapeutic potentials of lithium in treating brachial plexus injury.
